# Effects of resistance training on performance in competitive badminton players: a systematic review

**DOI:** 10.3389/fphys.2025.1548869

**Published:** 2025-06-05

**Authors:** Tao Wang, Ng Yee Guan, Saidon Amri, Tengku Fadilah Kamalden, Zhendong Gao

**Affiliations:** ^1^ Department of Sport Studies, Faculty of Educational Studies, University Putra Malaysia, Serdang, Selangor, Malaysia; ^2^ Department of Environmental and Occupational Health, Faculty of Medicine and Health Sciences, University Putra Malaysia, Serdang, Selangor, Malaysia; ^3^ National Sports Complex, Bukit Jalil, Kuala Lumpur, Malaysia

**Keywords:** resistance training, badminton, athletic performance, power, speed, agility, smash velocity

## Abstract

**Background:**

Resistance training (RT) is critical in enhancing athletic performance by improving physical fitness and sport-specific skills. However, there is a lack of systematic evaluation regarding the effects of RT on competitive athletes. This systematic review aims to explore the evidence of the impact of RT on physical performance and badminton-specific skills among competitive badminton players, providing actionable insights for optimizing training.

**Methods:**

Five databases—Web of Science, PubMed, Scopus, EBSCOhost, and SPORTDiscus—were systematically searched to identify studies investigating RT interventions for competitive badminton players. The search used a combination of keywords related to RT, physical fitness, and badminton. This review adhered to the Preferred Reporting Items for Systematic Reviews and Meta-Analyses (PRISMA 2020) guidelines.

**Results:**

RT interventions significantly improved power (e.g., countermovement jump, squat jump), speed (e.g., shuttle run), agility (e.g., directional changes), and endurance in competitive badminton players. Lower-limb RT demonstrated the strongest effects on jump performance, while core strength training enhanced technical stability and power transfer within kinetic chains. Upper-limb RT showed promising results in improving smash velocity and accuracy, though the evidence remains limited. Long-term interventions (>8 weeks) were more effective than shorter programs. Effective training modalities included plyometrics, eccentric overload, and functional core exercises.

**Conclusion:**

RT programs tailored to the physical and technical demands of badminton can significantly enhance athletic performance, particularly in high-intensity scenarios like offensive strokes and rapid directional changes. These findings highlight the importance of integrating RT into badminton-specific training regimens. Future research should focus on long-term RT effects and its impact on advanced skill execution.

**Systematic Review Registration:**

identifier CRD42024559831.

## 1 Introduction

Performance in badminton is determined by the interplay of speed, agility, flexibility, shoulder strength, explosive power, and muscular endurance, all of which are strongly correlated with playing ability (Cronin et al., 2001; Hughes et al., 2003; Hughes and Bopf, 2005; Jeyaraman et al., 2012; Singh J. et al., 2011; Singh NN. et al., 2011; Subramanian, 2013; Tiwari et al., 2011). The sport is characterized by repeated dynamic movements, including starts, stops, jumps, leaps, lunges, and rapid changes in direction (Lim et al., 2023), requiring a wide range of skilled postural adaptations and movement patterns (Malwanage et al., 2022). The velocity at which players strike the shuttlecock often serves as a critical determinant of success, underscoring the importance of strength training in enhancing this aspect of performance (Solanki and Gill, 2021). Achieving excellence in international competitions necessitates not only refined technical skills but also a substantial focus on physical conditioning (Cinthuja, 2015). Modern badminton increasingly prioritizes power and physical attributes, emphasizing aggressive offensive strategies to gain a competitive edge and secure victories.

Muscle strength is widely recognized as a fundamental factor in enhancing and maintaining athletic performance, including speed (Chelly et al., 2009), agility (Spiteri et al., 2015), and explosive power (Chelly et al., 2009; Andersen et al., 2010). Additionally, it plays a critical role in the development of motor performance (Suchomel et al., 2016). Resistance training (RT) is regarded as an effective approach to improving explosive power (Kraemer and Ratamess, 2004). RT can involve various muscle actions, including isometric (no net change in muscle length), isokinetic (constant movement velocity), and dynamic (a combination of eccentric and concentric actions), with dynamic RT being the most commonly utilized (American College of Sports Medicine, 2009). Initial improvements in muscle strength through RT are primarily attributed to neuromuscular adaptations, which enhance strength and functional performance (Pareja-Blanco et al., 2014). These adaptations include improved motor unit recruitment, increased synchronization, and a higher rate of force production, rather than muscle hypertrophy (Coyle et al., 1991). Hypertrophy-associated muscle adaptations further contribute to athletic performance, including increased anaerobic enzyme activity, enhanced force production, elevated intramuscular glycogen storage, and structural changes within primary muscle fiber types (Yamamoto et al., 2010).

RT is widely utilized by elite badminton players to enhance on-court performance (Sturgess and Newton, 2008). However, its application remains a topic of debate among coaches, who express concerns that increased muscle mass (hypertrophy) or reduced flexibility may create additional resistance, potentially hindering the performance of competitive badminton athletes. Despite these reservations, RT programs are frequently adopted by badminton players (Sturgess and Newton, 2008; Cui et al., 2024; Ihsan et al., 2024). The primary goal of RT methods is to overload the specific muscle groups used in competitive badminton, thereby enhancing maximum power output and improving performance during match play. The physiological benefits of RT are extensive, including increased phosphagen stores, contractile proteins, anaerobic power output, muscle structure, fiber pennation, protein synthesis, tissue remodeling, and hypertrophy of fast-twitch fibers (Goodwin and Cleather, 2016; Haff and Nimphius, 2012; Newton et al., 2011; Saltin and Gollnick, 2011). In badminton, athletes rely heavily on explosive power to execute technical movements, which is directly influenced by their muscle capacity (Crow et al., 2012). For badminton players, explosive power can be effectively enhanced through high-intensity RT.

To provide evidence-based recommendations for RT tailored to competitive badminton athletes and coaches, we conducted a systematic review of existing RT literature focusing on trained competitive badminton players. Although previous reviews have evaluated RT across multiple sports or among recreational athletes, few have specifically focused on competitive badminton players, leaving an important gap regarding tailored training interventions. Given the distinct physical and technical demands faced by elite badminton athletes, a comprehensive systematic review that consolidates RT evidence specifically for this population is critically needed. Coaches and elite badminton players frequently express concerns about potential adverse effects of RT, such as increased body mass or reduced flexibility, which could negatively impact badminton-specific performance. Previous studies often involved small sample sizes, short intervention durations, or lacked detailed methodologies, highlighting significant limitations in current evidence. Addressing these limitations is essential to clarify RT’s effectiveness and optimize training strategies specifically for competitive badminton players. Thus, this review systematically integrates and synthesizes the relevant literature, clearly addressing its applicability and implications for badminton performance enhancement. The objectives of this review are to (1) integrate existing literature to systematically examine the effects of RT methods on the physical fitness of competitive badminton players and (2) critically assess the impact of RT interventions on their badminton-specific technical performance.

## 2 Methods

### 2.1 Protocol and registration

The Eligibility Criteria of Preferred Reporting Items for Systematic Reviews and Meta-Analyses (PRISMA 2020) guidelines were followed in this systematic review (Page et al., 2021). This systematic review was registered with the International Prospective Register of Systematic Reviews (PROSPERO) on 20 June 2024 (Registration No.: CRD42024559831).

### 2.2 Eligibility criteria

This systematic review employs the PICOS model to establish inclusion and exclusion criteria for the literature (Table 1). The primary objective of this study is to evaluate the impact of RT on the performance of competitive badminton athletes. Studies were included if they met the following criteria: (1) Population: The study must focus on competitive-level badminton players, defined as professional athletes or individuals who have received formal coaching from recognized training institutions, sports academies, or reputable badminton clubs. Eligible participants include athletes of any gender, aged 10 years or older, with at least 3 years of training experience, or those who have competed in regional or higher-level competitions. (2) Intervention: The study must involve RT conducted independently and explicitly discussed, with a minimum intervention duration of 4 weeks. RT is defined as load-bearing or weighted activities, including exercises with free weights or machines. Subcategories include circuit training (a series of exercises performed in succession with minimal rest), heavy-load training (dynamic exercises such as squats and bench presses), and power training (weighted or stretch-shortening cycle exercises). (3) Study Design: Eligible studies include randomized controlled trials (RCTs), non-randomized controlled trials (nRCTs) with two or more groups, or single-group trials. (4) Outcomes: The study must report at least one effect of RT on the performance of competitive badminton players. Outcomes are categorized into physical performance and skill performance. Skill performance includes badminton-specific metrics such as smash speed, while physical performance encompasses components such as muscular strength, power, speed, coordination, endurance, flexibility, agility, stability, and balance.

**TABLE 1:** Inclusion criteria according to the PICOS condition.

Items	Detailed inclusion criteria
Population	Competitive-level badminton players
Intervention	Resistance training
Comparison	Two or more groups and single-group trials
Outcome	Physical fitness or badminton skill-related performance
Study designs	RCTs or nRCTs

RCTs, randomized controlled trials; nRCTs, non-randomized controlled trials.

The exclusion criteria were as follows: (1) Studies that do not involve competitive badminton players; (2) Studies where RT is not the primary intervention; (3) Reviews, editorials, or commentaries; (4) Non-English language studies, unless an English abstract is provided with sufficient detail to meet inclusion requirements; (5) Studies with incomplete data on outcomes of interest or those deemed to have insufficient methodological quality.

### 2.3 Information sources and search strategy

For this study, a comprehensive literature search was conducted using renowned national and international databases, including Web of Science, PubMed, Scopus, EBSCOhost, and SPORTDiscus. The search scope encompassed articles published in English from the inception of these databases up to 1 July 2024. To ensure comprehensive coverage, our research team also performed manual searches on Google Scholar and reviewed the reference sections of identified publications to include all relevant studies. The search terms used were: (“resistance training” OR “strength training” OR “weight training” OR “resistance exercise” OR “strength exercise” OR “resistance program” OR “strength program”) AND (“badminton” OR “badminton players” OR “competitive badminton” OR “elite badminton players” OR “badminton athletes”) AND (“performance” OR “athletic performance” OR “sports performance” OR “physical performance” OR “skill performance” OR “match performance”).

### 2.4 Study selection

This review utilized the Zotero reference management system to remove duplicates. Two authors (TW and NYG) independently screened the results based on titles and abstracts. Subsequently, two authors (TW and SA) reviewed these studies according to the inclusion criteria and PICOS framework. All processes were finalized through discussion, and any disagreements (e.g., intervention type, study design) were resolved through consultation with a third author (TF). Information extracted from the articles was entered into a Microsoft Excel spreadsheet (Microsoft Corporation, 2024) to assess inter-rater consistency throughout the PRISMA process (Narducci et al., 2011).

### 2.5 Data extraction

After selecting the studies, the authors (TW and NYG) extracted data including (1) author names and publication year; (2) population characteristics (age, gender, and competitive level of the badminton players); (3) primary area of intervention; (4) intervention details (type, duration, frequency, and intensity of resistance training); (5) comparison group (control or comparator group); (6) assessments (tests used to measure the effects of RT on athletes); and (7) outcomes (pre- and post-intervention results and between-group comparisons). The extracted information was entered into a Microsoft Excel spreadsheet (Microsoft Corporation, 2024), after which another author (GZ) reviewed the entries for accuracy.

### 2.6 Quality assessment

The 14-item “Qualsyst,” with specific criteria (yes = 2, partial = 1, no = 0), was employed to assess the quality of the studies (Kmet, 2004) (Table 2). This assessment tool was used in many reviews with topics like the present systematic review (Bravo et al., 2022; Cao et al., 2022; Cao et al., 2024). The quality of each included study was assessed independently by two authors (TW and NYG), and any discrepancies were discussed and resolved via consensus with a third author (SA). This tool categorized the selected studies into strong quality (75% or higher), moderate quality (55%–75%), and poor quality (less than 55%).

**TABLE 2:** Quality assessment of included studies using QualSyst framework.

Studies	Item number														Score	Rating
	Ⅰ	Ⅱ	Ⅲ	Ⅳ	Ⅴ	Ⅵ	Ⅶ	Ⅷ	Ⅸ	Ⅹ	XI	XII	XIII	XIV		
Sun and Shao (2023)	2	2	1	2	2	0	0	2	1	2	2	2	2	2	22	Strong
Nirendan (2023)	2	2	2	1	2	0	0	2	1	2	2	1	2	2	21	Strong
Chansrisukot et al. (2015)	2	2	1	2	2	0	0	2	1	2	2	1	2	2	21	Strong
Huang et al. (2023)	2	2	1	2	2	0	0	2	1	2	2	1	2	2	21	Strong
Andersen et al. (2007)	2	2	2	2	0	0	0	2	2	2	2	1	2	2	21	Strong
Yisi (2023)	2	2	1	2	2	0	0	2	1	2	2	1	2	2	21	strong
Biao and Lu (2023)	2	2	1	2	2	0	0	2	1	2	2	1	2	2	21	strong
Yüksel and Akın (2017)	2	2	1	2	2	0	0	2	1	2	2	1	2	2	21	strong
Jianping (2021)	2	1	1	1	0	0	0	2	1	2	2	1	2	2	18	Moderate
Fröhlich et al. (2014)	2	2	1	2	0	0	0	2	1	2	2	1	2	2	19	Moderate
Wiriawan et al. (2024)	2	1	1	2	0	0	0	2	1	2	2	1	2	2	18	Moderate
Sawant (2023)	2	2	2	1	2	0	0	2	1	2	2	1	2	2	19	Moderate
Low et al. (2023)	2	2	2	2	2	0	0	2	1	2	2	1	2	2	20	Moderate
Middleton et al. (2016)	2	2	1	2	0	0	0	2	1	2	2	1	2	2	19	Moderate

2 indicates yes, 1 indicates partial, 0 indicates no, I question described, II, appropriate study design; III, appropriate subject selection; IV, characteristics described, V random allocation, VI, researchers blinded; VII, subjects blinded; VIII, outcomes measure well defined and robust to bias; IX, sample size appropriate, X analytic methods well described, XI, estimate of variance reported; XII, controlled for confounding; XIII, results reported in detail, and XIV, conclusion supported by results.

### 2.7 Data synthesis

The included studies demonstrated insufficient homogeneity in terms of participant characteristics, intervention protocols, and outcome measures (Deeks et al., 2019). Notably, the studies did not consistently provide three or more baseline and follow-up measurements for identical variables. As a result, a narrative synthesis of the findings from the included studies was conducted (Table 3). The interventions identified were either RT alone or RT in combination with other exercise modalities. The extracted data were analyzed in accordance with the recommendations of the Centre for Reviews and Dissemination (Akers et al., 2009).

**TABLE 3:** Characteristics and outcomes of studies on resistance training effects in competitive badminton players.

Study	Participants (N, age, level, experience)	Training focus (training modalities)	Intervention details	Comparator	Outcome measures	Outcome
Sun and Shao (2023)	N: 20 M; A: EG: 18.2 ± 0.7 years, CG: 17.8 ± 1.3 years; L: Sports College players; TE: NR	Core (sit-ups, planks, stability ball exercises)	Freq: 3 sessions (25 min)/week; Length: 10 weeks	EG: progressive core strength training CG: NBT	FMS (Squat, hurdles, Straight Lunge, shoulder flexibility, Lower waist flexibility, body control push up, swivel stability); Technical Performance (MBT, badminton throw, backhand and forehand performance)	FMS ↑ (EG), ↔ (CG); Technical Performance ↑ (EG), ↔ (CG)
Nirendan (2023)	N: 30 M; A: 18–25 years; L: Club players; TE: NR	Core (plank variations, burpees)	Freq: 3 sessions (45 min)/week; Length: 12 weeks	EG: core strength training CG: NBT	Muscular strength (Plank Test), Muscular endurance (Burpee Test)	Muscular Strength ↑ (EG, P < 0.05), ↔ (CG); Muscular Endurance ↑ (EG, P < 0.05), ↔ (CG)
Yüksel and Akın (2017)	N: 22 M, 18 FM; A: 16–24 years (SD = 1.92); L: National players; TE: 9.13 ± 1.87 years	Core (star balance training, dynamic core drills)	Freq: 3 sessions (20–25 min)/week; Length: 8 weeks	EG: Core Strength Training CG: NBT	Dynamic balance: SEBT	SEBT ↑ (EG, P < 0.01); ↔ (CG)
Chansrisukot et al. (2015)	N: 40 M; A: 14–18 years; L: Professional players; TE: NR	Lower body (deep squats, jump squats, reaction drills)	Freq: 3 sessions (30 min)/week; Length: 8 weeks	EG1: CPT + NBT EG2: EPT + NBT EG3: CPT + EPT + NBT CG: NBT	Reaction time (RT), Movement time (MT), Response time (RP)	RT ↑ (CPT, CPT+EPT), ↔ (EPT, CG); MT ↑ (EPT, CPT+EPT), ↔ (CPT, CG); RP ↑ (EPT, CPT+EPT), ↔ (CPT, CG)
Huang et al. (2023)	N: 11 M, 7 FM; A: 21.4 ± 1.4 years; L: Collegiate players players; TE: ≥3 years	Lower body (APRE back squats, velocity-based loads)	Freq: 2 sessions (120 min)/week; Length: 4 weeks	APRE: 6 RM back squats VBRT: velocity-based loads	Jump performance (CMJ, SJ, DJ), EUR, RSI	CMJ ↑ (APRE P = 0.04), ↔ (VBRT, P > 0.05); SJ ↔ (APRE, VBRT); EUR ↔ (APRE, VBRT); RSI ↑ (APRE, P = NS); ↔ (VBRT, P > 0.05)
Yisi (2023)	N: 24; A: EG: 20.717 ± 0.7293 years, CG: 20.411 ± 0.7900 years; L: Professional player; TE: ≥4 years	Lower body (resistance band drills, directional runs)	Freq: 3 sessions/week; Length: 6 weeks	EG: lower-limb resistance training CG: NBT	SJ, SLJ, 30 m Running, 4 x 10 m Running, Directional Tests (LRMT, FBMT, Low-Gravity Four-Point Run Time, DJHT, TRBS)	SJ ↑ (EG), ↔ (CG); SLJ ↑ (EG), ↔ (CG); 30 m ↑ (EG), ↔ (CG); 4 * 10 m ↑ (EG), ↔ (CG); Directional Tests ↑ (EG), ↔ (CG)
Jianping (2021)	N: 20 M; A: NR; L: Collegiate players; TE: NR	Lower body (weighted step-ups, single-leg agility drills)	Freq: 3 sessions (90 min)/week; Length: 8 weeks	Single group (lower limb strength and agility training)	Strength (SLJ), Agility (10-Meter Round-Trip)	SLJ ↑ (7.95 cm, P < 0.01), 10-Meter Round-Trip ↑ (0.465 s, P < 0.01)
Fröhlich et al. (2014)	N: 8 M, 3FM; A: 16.0 ± 1.6; L: National players; TE: NR	Lower body (box jumps, drop jumps)	Freq: 2 sessions (30 min)/week; Length: 8 weeks	Single group (plyometric training)	SJ, CMJ, DJ, 2D Video Analysis	All ↑
Wiriawan et al. (2024)	N: 23 M; A: 16–20 years (SD = 1.16); L: Club players; TE: 8.04 years (SD = 1.07)	Lower Body (swiss ball hamstring curl, single-leg bridge)	Freq: 3 sessions/week; Length: 10 weeks	Single Group (Swiss ball hamstring curl and single leg bridge)	Norbord Test (Hamstring Asymmetry), CMJ	Hamstring Asymmetry ↓ (-11.06%, Cohen's d = 1.443, P ≤ 0.001); CMJ ↑ (+2.08 cm, Cohen's d = 0.447, P ≤ 0.001)
Sawant (2023)	N: 2 M; A: 17-19; L: Club players; TE: NR	Lower body (vertical jump drills, medicine ball throws)	Freq:4 sessions (90 min)/week; Length: 6 weeks	EG: Plyometric exercises CG: NBT	Explosive Power (VJ, MBT)	VJ ↑ (EG); ↔ (CG); MBT ↑ (EG); ↔ (CG)
Low et al. (2023)	N: 36; A: 20.6 ± 1.2 years; L: Collegiate players; TE: ≥4 years	Lower Body (Flywheel Squats)	Freq:2 sessions/week; Length: 4 weeks	EG: FEO PT: Plyometric exercises CG: NBT	Explosive Power (CMJ, RSI), Agility (BAT), Flexibility (Sit-and-reach test)	CMJ ↑ (FEO/PT, P < 0.001), ↔ (CG); RSI ↑ (FEO, PT), ↔ (CG); BAT ↑ (FEO/PT, P < 0.001), ↔ (CG); Sit-and-reach test ↔ (FEO, PT, CG)
Biao and Lu (2023)	N: 24 M; A: EG: 22.10 ± 1.568 years CG: 21.61 ± 1.228 years; L: Club players; TE: NR	Lower body + core (rope jumps, weighted lunges)	Freq: NR sessions (40 min)/week; Length: 12 weeks	EG: high-load strength training CG: NBT	30 s Rope Jump, 1 Min Sit-ups, 1 Min Prone from Both Ends, Badminton Throw, Straight Turn Back Run, Low Center of Gravity Corner Run, LRMT, SV (Operating Speed, Release Speed, Net Speed, Landing Time)	30 s rope jump ↑ (EG), ↔ (CG); 1 min sit-ups ↑ (EG, CG); 1 min prone ↑ (EG), ↔ (CG); Badminton throw ↑ (EG), ↔ (CG); Straight turn back run ↑ (EG), ↔ (CG); Low center of gravity corner run ↑ (EG), ↔ (CG); LRMT ↑ (EG), ↔ (CG); SV ↑ (EG, +15%, P < 0.01), ↔ (CG)
Andersen et al. (2007)	N: 70 M; A: EG: 23.5 ± 3.5 years, CG: 23.2 ± 1.9 years; L: National players (EG); TE: 7.5 ± 3.5 years	Lower body + core (high-load squats, plank variations)	Freq: 3 sessions/week; Length: 14 weeks	EG (Badminton group): Resistance Training CG (Reference group): Resistance Training	Knee Extensors/Flexors Torque, RFD	Knee Extensors Torque ↑ (EG), ↔ (CG); Flexors Torque ↑ (EG), ↔ (CG); RFD ↑ (EG, P < 0.05), ↔ (CG)
Middleton et al. (2016)	N: 1 M; A: 18 years; L: National player; TE: NR	Upper body + lower body (medicine ball throws, box drills)	Freq: 1 session (60 min)/week; Length: 8 weeks	RPT (Case study)	Jump performance (CMJ, SLJ), Upper Body Power (1-MBT, 2-MBT), Speed (5 m and 10 m sprints), Agility (Sideways AT, Four-Corner AT)	CMJ ↑, SLJ ↑, 1-MBT ↑, 2-MBT ↑, 5 m and 10 m sprints ↑ (−0.12 s, P = NS), Sideways AT ↑ (−0.39 s, P = NS), Four-Corner AT ↔

A, age; FM, female; M, male; NR, not reported; L: level; TE, training experience; EG, experimental group; CG, control group; FMS, motor function screen; MBT, Medicine Ball Throw (1-MBT: One-Arm, 2-MBT: Two-Arm); EPT, explosive power training; CPT, cognitive psychological training; NBT, normal badminton training; APRE, autoregulatory progressive resistance exercise; VBRT, velocity-Based Resistance Training; CMJ, countermovement jump; SJ, squat jump; DJ, drop jump; EUR, eccentric utilization ratio; RSI, reactive strength index; RPT, resistance and plyometric training; SLJ, standing long jump; AT, agility test; LRMT, left and right movement time; FBMT, forward and backward movement time; DJHT, drill and jump hurdle test; TRBS, touch and run between sidelines; SV, smash velocity; SEBT, star excursion balance test; RFD, rate of force development; VJ, vertical jump; FEO, flywheel eccentric overload training; BAT, bandcamp agility test; ↑, Increase; ↓, Decrease, ↔ (NS), no significant change; *P* < 0.05, Statistically significant improvement.

## 3 Results

### 3.1 Study selection

**FIGURE 1:**
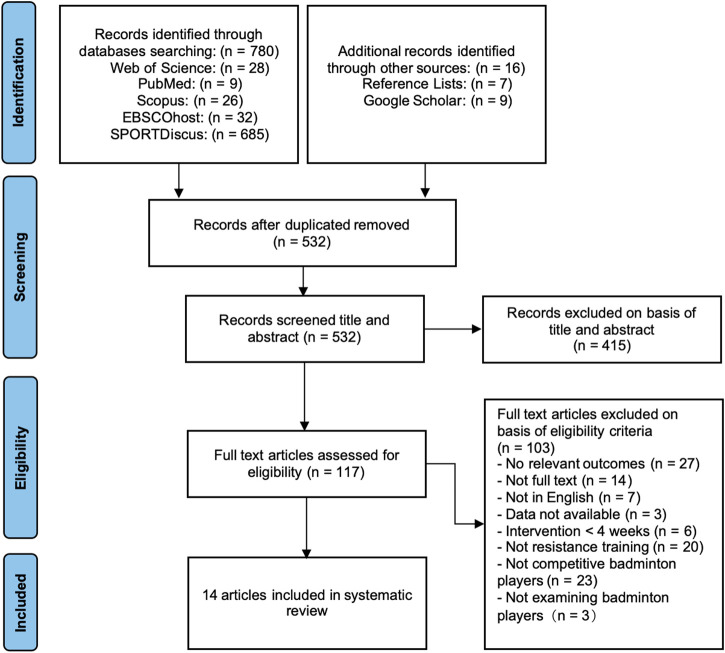
PRISMA flow diagram.

As illustrated in Figure 1 (PRISMA Flowchart), a total of 780 articles were identified across five databases, with an additional 16 studies retrieved through manual searches on Google Scholar and reference lists. These searches specifically targeted articles assessing the athletic performance of competitive badminton players in response to RT. All articles were systematically reviewed, and relevant data were recorded. Reference lists of identified studies were also examined to ensure the inclusion of additional eligible studies. Many articles did not meet the inclusion criteria—such as review articles or training studies focusing on untrained or recreationally active participants—and were excluded from the analysis. However, these excluded articles were retained for contextual review and discussion. Ultimately, 14 articles met the eligibility criteria and were included in the quantitative synthesis (Figure 1).

### 3.2 Study quality assessment

The quality of the 14 included studies was independently assessed by two authors using the “QualSyst” tool. Among these, eight studies were rated as high-quality (Sun and Shao, 2023; Nirendan, 2023; Chansrisukot et al., 2015; Huang et al., 2023; Andersen et al., 2007; Yisi, 2023; Biao and Lu, 2023; Yüksel and Akın, 2017), while the remaining six studies were rated as moderate quality (Jianping, 2021; Fröhlich et al., 2014; Wiriawan et al., 2024; Sawant, 2023; Low et al., 2023; Middleton et al., 2016). No studies were excluded based on the quality assessment.

### 3.3 Participant characteristics

The population characteristics of the 14 studies were reported based on the following (Table 3):

(1) Sample Size. 377 participants were included across all studies, with sample sizes ranging from 1 (Middleton et al., 2016) to 70 participants (Andersen et al., 2007). The mean sample size was 27 participants (SD = 18.7), reflecting a mixture of small-scale interventions and larger cohort studies.
(2) Sex. Nine studies exclusively investigated male participants (Sun and Shao, 2023; Nirendan, 2023; Chansrisukot et al., 2015; Andersen et al., 2007; Yisi, 2023; Biao and Lu, 2023; Wiriawan et al., 2024; Sawant, 2023; Middleton et al., 2016). Three studies included mixed-gender populations (Huang et al., 2023; Yüksel and Akın, 2017; Fröhlich et al., 2014), while two studies did not report the sex of participants (Jianping, 2021; Low et al., 2023).
(3) Age. The participants’ ages ranged from 14 (Chansrisukot et al., 2015) to 25 years (Nirendan, 2023), with most studies focusing on late adolescents and young adults. For studies that reported mean age, values ranged from 17.8 ± 1.3 years (Sun and Shao, 2023) to 23.5 ± 3.5 years (Andersen et al., 2007).
(4) Level. Six studies recruited participants with a professional or club-level background (Nirendan, 2023; Chansrisukot et al., 2015; Yisi, 2023; Biao and Lu, 2023; Wiriawan et al., 2024; Sawant, 2023). Four studies focused on elite university athletes (Sun and Shao, 2023; Huang et al., 2023; Jianping, 2021; Low et al., 2023), while another four studies investigated participants with national-level experience (Andersen et al., 2007; Yüksel and Akın, 2017; Fröhlich et al., 2014; Middleton et al., 2016).
(5) Training Focus. The included studies addressed a wide range of training areas: Three studies focused on core strength training (Sun and Shao, 2023; Nirendan, 2023; Yüksel and Akın, 2017). Eight studies investigated lower-limb RT (Chansrisukot et al., 2015; Huang et al., 2023; Yisi, 2023; Jianping, 2021; Fröhlich et al., 2014; Wiriawan et al., 2024; Sawant, 2023; Low et al., 2023). Two studies explored the effects of combined lower-limb and core RT (Andersen et al., 2007; Biao and Lu, 2023), while only one study investigated the combination of upper- and lower-limb training (Middleton et al., 2016). Interestingly, none of the studies targeted upper-limb RT exclusively.

### 3.4 Intervention characteristics

The characteristics of the included studies were as follows:

(1) Training Program Length: The training program length varied across studies, ranging from 4 weeks (Huang et al., 2023; Low et al., 2023) to 14 weeks (Andersen et al., 2007), with a mean training program length of approximately 8.9 weeks (SD = 3.2). Most studies implemented interventions lasting between 6 and 10 weeks, reflecting the typical duration for RT studies targeting specific physical or technical adaptations.
(2) Training Duration: Each training session was reported in ten studies. Session durations ranged from 20 to 25 min (Sun and Shao, 2023; Yüksel and Akın, 2017) to 120 min per session (Huang et al., 2023). However, four studies did not specify session duration (Andersen et al., 2007; Yisi, 2023; Wiriawan et al., 2024; Low et al., 2023).
(3) Training Frequency: Thirteen studies detailed the frequency of training sessions per week, which ranged from 1 (Middleton et al., 2016) to 4 times per week (Sawant, 2023). The most common frequency was 3 times per week, reported in eight studies (Sun and Shao, 2023; Nirendan, 2023; Chansrisukot et al., 2015; Yisi, 2023; Biao and Lu, 2023; Yüksel and Akın, 2017; Jianping, 2021; Wiriawan et al., 2024). Only one study (Biao and Lu, 2023) did not specify training frequency.
(4) Training Modalities: The included studies investigated diverse training modalities, which can be categorized as follows:
  a) Core Strength Training: This training focuses on improving core stability, balance, and overall body control, which are critical for generating power and maintaining stability during rapid directional changes in badminton (Sun and Shao, 2023; Nirendan, 2023; Yüksel and Akın, 2017). Interventions included progressive core stability exercises such as planks, dynamic balance drills, and body control push-ups.
  b) Flywheel Eccentric Training: This training targets the enhancement of lower-limb explosive power through eccentric overload, emphasizing controlled eccentric movements that benefit jumping and rapid acceleration in badminton (Low et al., 2023). Interventions included flywheel squats and other eccentric overload exercises.
  c) Single-Leg Strength Training: This training focuses on developing unilateral lower-limb strength and balance, which are essential for performing lunges and rotational movements in badminton (Jianping, 2021; Wiriawan et al., 2024). Interventions included Swiss ball hamstring curls and single-leg squats.
  d) Lower-Limb Plyometric Training: This training aims to enhance lower-limb power and reactive strength, both of which are crucial for executing quick jumps and rapid directional changes in badminton (Chansrisukot et al., 2015; Fröhlich et al., 2014; Sawant, 2023). Interventions included countermovement jumps (CMJ), squat jumps (SJ), and drop jumps (DJ).
  e) Combined Lower-Limb and Core RT: This training integrates core stability and lower-limb strength exercises to improve athletes’ balance and power (Andersen et al., 2007; Biao and Lu, 2023). The intervention involved high-load strength training conducted over 12–14 weeks, incorporating exercises such as squats and sit-ups.
  f) Upper- and Lower-Limb Training: This combined training approach is designed to enhance jumping ability, agility, and power (Middleton et al., 2016). Interventions included resistance and weighted exercises, such as medicine ball throws.
  g) Cognitive Psychological Training Combined with Explosive Power Training: This training integrates cognitive-perceptual tasks with explosive power exercises to simultaneously improve reaction speed, movement time, and explosive strength (Chansrisukot et al., 2015). Interventions included weighted exercises such as squat jumps and countermovement jumps to enhance explosive power.

### 3.5 Outcome characteristics

#### 3.5.1 Effect of RT on power

Six studies examining jump performance (Huang et al., 2023; Yisi, 2023; Fröhlich et al., 2014; Wiriawan et al., 2024; Sawant, 2023; Low et al., 2023) reported significant improvements in countermovement jump (CMJ), squat jump (SJ), and vertical jump (VJ) outcomes. Flywheel eccentric overload training (Low et al., 2023) and weight-loaded training (Fröhlich et al., 2014; Sawant, 2023) were particularly effective. Additionally, two studies (Sun and Shao, 2023; Sawant, 2023) emphasized upper-body power, demonstrating significant improvements in medicine ball throw (MBT) performance following core and weight-loaded training interventions. These findings underscore the substantial impact of RT on enhancing power, particularly in lower-limb explosive movements.

#### 3.5.2 Effect of RT on muscle strength

Improvements in muscle strength were particularly evident in studies focusing on core and lower-limb RT. Two studies (Andersen et al., 2007; Biao and Lu, 2023) reported significant gains in knee extensor and flexor strength following high-load strength training. Another study (Nirendan, 2023) demonstrated improvements in core strength and overall muscular strength as measured by plank tests. Although upper-limb RT was less frequently investigated among the selected studies, one study (Middleton et al., 2016) reported notable enhancements in both one-arm and two-arm medicine ball throw (MBT) performance.

#### 3.5.3 Effect of RT on speed

Speed performance in the selected studies was evaluated through sprint speed and movement velocity. Two studies (Yisi, 2023; Sawant, 2023) reported significant improvements in 30-m and 5–10 m sprint times within the experimental groups. Additionally, two other studies (Biao and Lu, 2023; Low et al., 2023) documented enhanced agility-related speed performance, including improvements in the Bandcamp Agility Test (BAT) and lateral movement times.

#### 3.5.4 Effect of RT on agility

Six studies reported improvements in agility following RT interventions. Among these, two studies employed directional agility tests, such as shuttle runs (Yisi, 2023) and directional movement drills (Biao and Lu, 2023), both demonstrating significant enhancements after lower-limb resistance and weight-loaded training. Agility gains in the 10-m round-trip test were observed following weighted step-ups and single-leg agility drills (Jianping, 2021). Significant improvements in Star Excursion Balance Test (SEBT) scores were reported, reflecting enhanced stability during dynamic movements (Yüksel and Akın, 2017). Performance gains in the Bandcamp Agility Test (BAT) were linked to flywheel eccentric overload training and plyometric exercises (Low et al., 2023). Lastly, agility improvements in badminton-specific tests, such as the Sideways Agility Test and the Four-Corner Agility Test, were demonstrated following a combined upper- and lower-limb RT program (Middleton et al., 2016).

#### 3.5.5 Effect of RT on endurance

Among the selected studies, few explicitly measured endurance outcomes. One study focusing on muscular endurance (Nirendan, 2023) reported improved burpee test performance following core RT. Another study (Biao and Lu, 2023) emphasized functional endurance, using rope jump performance as a specific measure of badminton-related endurance, which showed significant improvements.

#### 3.5.6 Effect of RT on badminton-related skills

Three studies examined the effects of RT on badminton-related skills. Improvements in smash velocity and shuttle speed were observed in one study (Biao and Lu, 2023). Another study reported increases in forehand and backhand accuracy and strength following progressive core strength training (Sun and Shao, 2023). Additionally, reductions in reaction time and improvements in movement efficiency were observed after combined cognitive and explosive power training (Chansrisukot et al., 2015). These findings suggest that RT can significantly enhance badminton-specific skill performance in competitive players.

## 4 Discussion

### 4.1 Methodological considerations

To critically evaluate the findings of this review, several methodological factors must be considered. The included studies demonstrated significant variability in sample sizes, participant characteristics, and intervention designs. Most studies had relatively small sample sizes (Sun and Shao, 2023; Wiriawan et al., 2024), which limits the generalizability of findings and the strength of conclusions drawn regarding RT effectiveness for diverse competitive badminton athletes. Additionally, although most studies reported positive outcomes from RT interventions, many were of relatively short duration, which restricted the ability to assess long-term adaptations effectively.

Notably, previous research in other sports, such as cycling and basketball, has demonstrated that RT interventions lasting longer than 8 weeks lead to more sustained performance improvements (Crowley et al., 2017; Ronnestad et al., 2008). These findings highlight the importance of longer-duration RT interventions in badminton, yet only a few studies in our review adopted extended intervention periods (Huang et al., 2023; Low et al., 2023). This limitation underscores the need for future research to include longer-duration RT programs to validate long-term effectiveness explicitly.

Moreover, only a few studies implemented RT programs during competitive seasons, which poses unique challenges for athletes (Low et al., 2023). The training phase and specific RT protocols are critical considerations in designing effective periodized RT programs. Future studies should clearly define the timing of RT interventions within the athletes’ competitive seasons to enhance practical relevance and implementation.

Future research should also prioritize standardized training protocols, consistent outcome measurements, and advanced monitoring methods, such as velocity-based resistance training (VBRT) feedback (Zhang et al., 2022), to facilitate more precise evaluations of RT interventions.

### 4.2 Effects of RT on power

Research has demonstrated that RT significantly enhances power, particularly in jump-related movements. Studies focusing on lower-limb exercises, such as plyometric training and eccentric overload, have reported substantial improvements in countermovement jump (CMJ), squat jump (SJ), and vertical jump (VJ) performance (Huang et al., 2023; Fröhlich et al., 2014; Sawant, 2023; Low et al., 2023). For instance, the effectiveness of autoregulatory progressive resistance exercise (APRE) in improving explosive power among collegiate badminton players was emphasized (Huang et al., 2023).

Explosive power is especially critical in badminton, where a significant proportion of technical actions—such as jumping smashes and rapid directional changes—depend on this physical capacity (Crow et al., 2012). Enhanced explosive power, closely linked to muscle strength, provides athletes with a competitive edge during match play. However, further research is required to clarify whether these gains translate effectively into competitive match performance, where additional factors such as fatigue, psychological stress, and tactical demands are present.

Comparatively, plyometric training may rely more on technical execution and optimization of the stretch-shortening cycle (Meylan and Malatesta, 2009), highlighting the importance of tailored interventions based on athletes’ technical proficiency and training experience. Similarly, studies have shown that flywheel eccentric overload (FEO) training significantly increases jump height and power output, particularly for less experienced athletes, due to its lower technical demands (Low et al., 2023; Suchomel et al., 2021). These findings are consistent with previous research in other sports, emphasizing the critical role of eccentric overload and high-intensity weight-loaded exercises in power development.

Upper-limb power is equally vital in badminton, especially for offensive actions like smashes. A direct correlation between upper-limb explosive power and smash velocity in badminton players was identified, demonstrating that greater upper-limb explosive power is significantly associated with higher smash velocity and improved accuracy (Indora et al., 2022). Similarly, a strong relationship between arm muscle explosive power and smash performance was reported, reinforcing the importance of targeted arm muscle training to enhance offensive capabilities in competitive play (Pratama, 2020).

Biomechanical analyses have further highlighted the contributions of specific upper-limb movements to smash effectiveness. Wrist movement has been identified as a key determinant of smash efficiency compared to elbow and shoulder movements, suggesting that targeted wrist training should be a focus in upper-limb RT (Shan et al., 2015). Additionally, the explosive strength of the arms has been shown to significantly enhance the power and effectiveness of smashes, particularly in junior players, making focused upper-limb training a critical component of overall RT programs (Syafriandi, 2020).

Finally, improvements in medicine ball throw (MBT) performance were reported following core strength training, further emphasizing the role of RT in enhancing explosive power (Sun and Shao, 2023). These findings suggest that RT, when tailored to the specific demands of badminton, is an effective strategy for improving high-performance actions, including jumping smashes and rapid directional changes, which are essential for competitive success.

### 4.3 Effect of RT on muscle strength

RT is recognized as one of the most effective methods for assessing and developing strength and explosiveness in athletes (Fry, 2004). It is also the most widely used exercise intervention for increasing muscular strength (American College of Sports Medicine, 2009). RT can be performed using isometric muscle actions (i.e., with no net change in muscle length), isokinetic muscle actions (i.e., with a constant rate of movement), and the most selected, dynamic muscle actions (i.e., coupled eccentric and concentric actions) (American College of Sports Medicine, 2009). The neuromuscular system adapts explicitly to the stimuli it is exposed to, leading to increased muscle strength and functional performance (Pareja-Blanco et al., 2014).

In all the included studies, improvements in muscle strength were evident, particularly in interventions focusing on the lower limbs and core. Incorporating lower-limb RT, such as resistance band exercises, into regular badminton practice has been shown to significantly enhance muscular strength and explosive power in the lower limbs. This approach improves training efficiency, enabling athletes to achieve better results in a shorter time and enhancing performance and competitiveness in matches (Yisi, 2023). Additionally, studies have demonstrated that high-load RT significantly enhances knee extensor and flexor strength (Andersen et al., 2007; Biao and Lu, 2023).

Progressive core training has been shown to improve trunk stability and overall strength (Nirendan, 2023). In badminton, enhanced core strength contributes to greater stability during technical movements and improved efficiency of the kinetic chain (Yaprak and Küçükkubaş, 2020). For example, during a jump smash, the lower limbs and core muscles generate force against the ground, transferring power through the kinetic chain to the upper limbs (Oliva-Lozano and Muyor, 2020). Athletes with stronger core strength exhibit faster force transmission, leading to higher-quality smashes (Sun and Shao, 2023). Future research should explore whether improvements in core strength directly translate to sustained skill performance enhancements under competitive conditions.

Although studies on upper-limb RT were limited among the selected literature, positive effects were still observed. Improvements in medicine ball throw (MBT) performance were reported (Middleton et al., 2016). Additionally, research indicates that for performance-oriented elite badminton players, a suitable time for high-intensity upper-limb RT is during the preparatory phase, typically scheduled a few months before the competitive season. This phase establishes the physical fitness foundation required to support the subsequent competitive season, including multiple seasonal peaks. Following strength-building RT, plyometric or functional training can be introduced, with the training concluding 1–2 weeks before competition to allow players sufficient recovery time to transition into the competitive phase (Fröhlich et al., 2014).

These findings are consistent with observations highlighting that incorporating weight-bearing exercises into regular training regimens significantly enhances muscular strength (Liu and Liu, 2023). The content and duration of RT programs play a critical role, with programs exceeding 8–12 weeks and incorporating structured progression yielding superior outcomes. Nevertheless, the limited evidence on upper-limb RT emphasizes the need for more targeted research in this area.

### 4.4 Effects of RT on speed and agility

Badminton is rated as an ultra-fast game, giving faster players an advantage over slower competitors (Sturgess and Newton, 2008; Bańkosz et al., 2013). However, training players for faster movement remains a challenge for coaches (Colfer, 1977). Raw speed alone does not guarantee victory in badminton (Rambely et al., 2005). Success relies on moving accurately and quickly, with laser-point precision, while executing proper techniques and tactics (Kuntze et al., 2010).

Speed and agility, as fundamental elements for success in badminton, are positively influenced by RT interventions. Improved sprint times for 30-m and 5–10-m distances have been observed following plyometric and lower-limb RT interventions (Yisi, 2023; Sawant, 2023). Additionally, agility-focused drills demonstrated enhanced directional movement times (Biao and Lu, 2023; Low et al., 2023). These findings support the established link between explosive power training and reduced movement time (Chansrisukot et al., 2015; Rhea et al., 2008).

Notably, cognitive-perceptual training combined with RT further improved reaction time and movement efficiency, highlighting the value of integrating cognitive elements into physical training (Chansrisukot et al., 2015). This approach aligns with findings emphasizing that precise movement execution is as critical as speed in badminton (Rambely et al., 2005). Future studies should investigate more thoroughly how cognitive-perceptual RT interventions can be effectively integrated within periodized training plans to optimize competition performance.

### 4.5 Effects of RT on endurance

Endurance outcomes, though less frequently assessed, indicated meaningful improvements in muscular and functional endurance. Core training interventions enhanced muscular endurance, as evidenced by improved burpee test scores (Nirendan, 2023). Similarly, rope jump performance, used as a proxy for badminton-specific endurance, showed significant gains following lower-limb RT (Biao and Lu, 2023). These findings align with observations highlighting the dual demands of aerobic and anaerobic endurance in competitive badminton (Andersen et al., 2007). However, further research into the optimal frequency and duration of RT for endurance development remains warranted.

### 4.6 Effects of RT on Balance and Flexibility

Balance and flexibility are critical for executing rapid directional changes and maintaining stability during play. Core strength training has been shown to effectively enhance dynamic balance, with significant improvements observed in metrics such as the Star Excursion Balance Test (SEBT) after 8 weeks of core training (Sun and Shao, 2023).

Flexibility, however, was rarely assessed in the included studies. Changes in movement direction and reactions to shuttle placement require athletes to respond and move within less than one second to return the shot, making agility a crucial fitness component in badminton (Chen et al., 2009). Additionally, flexibility is vital for covering all areas of the court during reaching, diving, and lunging for the shuttle (Cinthuja, 2015). Future research should explicitly address both balance and flexibility, exploring their mechanisms and interactions within integrated RT programs to further optimize badminton-specific performance.

### 4.7 Effects of RT on badminton-related skills

Research has demonstrated that RT positively impacts badminton-specific skills, particularly smash velocity, shuttle speed, and stroke accuracy. Significant improvements in smash velocity were observed following progressive upper-limb strength training (Biao and Lu, 2023), while core strength training was associated with enhanced stroke accuracy and power (Sun and Shao, 2023). Additionally, reductions in reaction and movement times were noted after cognitive and explosive power training, emphasizing the role of RT in improving reaction capabilities and technical efficiency during gameplay (Chansrisukot et al., 2015). These findings highlight the importance of RT in enhancing technical stability and kinetic chain efficiency, both of which are critical for high-intensity badminton actions. Nevertheless, more standardized and detailed methodologies are required to clearly delineate the direct relationship between RT interventions and skill improvements in competitive match settings.

The jump smash is widely regarded as the most aggressive stroke in badminton, placing additional physical demands on players, including speed, power, precision, flexibility, and coordination (Clement, 2014; Ramasamy et al., 2021). Compared to the standing smash, the jump smash offers a higher contact point, steeper trajectory, and faster shuttle speed, making it more difficult for opponents to return (Phomsoupha and Laffaye, 2015). However, executing a high-quality jump smash requires considerable physical exertion, highlighting the need for targeted training programs to optimize performance.

Studies have shown a strong correlation between upper-limb strength and smash performance, which is crucial in competitive badminton. For example, elite Malaysian players exhibit superior upper-limb strength compared to sub-elite athletes, particularly in the 1-RM bench press, enabling them to generate greater shuttle velocity—a key determinant of smash effectiveness (Ooi et al., 2009). Similarly, the significant relationship between racket velocity and shoulder internal rotation torque highlights the importance of angular-specific strength training for improving smash performance (Awatani et al., 2018).

RT can also improve other badminton-related skills, such as forearm strength, which is essential for doubles players in jabbing and pushing movements. A positive correlation between arm muscle strength and smash performance has been identified, emphasizing that explosive arm action serves as the foundation for powerful smashes (Sakurai and Ohtsuki, 2000). Specifically, the bench press contributes to shoulder internal rotation and adduction, as well as elbow extension—all critical movements in the smash motion (Sakurai and Ohtsuki, 2000). These findings indicate that targeted RT, including bench press exercises, can effectively enhance both offensive and defensive capabilities in badminton players.

Body positioning plays a crucial role in stroke effectiveness, with proper placement relative to the shuttlecock significantly influencing both power and accuracy (Dai et al., 2009). Furthermore, power and accuracy have been identified as essential parameters for evaluating the effectiveness of sports skills (Ambre, 2023). These findings underscore the importance of integrated RT programs that enhance athletes’ physical readiness, enabling dynamic adjustments in body positioning during high-pressure match scenarios. Such strategies align with the strategic integration of RT into the training regimens of the Chinese national badminton team to optimize competitive performance (Li, 2016).

In conclusion, RT programs tailored to the technical and physical demands of badminton can significantly improve skill performance, particularly in high-intensity scenarios such as offensive strokes and rapid directional changes. However, gaps remain in understanding the long-term effects of such interventions on elite competitive performance. Future research should focus on badminton-specific outcomes, such as forehand jump smash precision and agility-driven shot accuracy, to further optimize training strategies for elite players and align results more closely with the sport’s unique demands.

### 4.8 Implications and future directions

This review highlights the multifaceted benefits of RT for badminton players, encompassing physical improvements in power, strength, and agility, as well as sport-specific skill enhancements. However, several gaps in the current literature must be addressed to further optimize RT programs for competitive badminton.

A critical gap identified is the lack of targeted research on upper-limb strength. Upper-limb strength and power are essential for executing high-speed smashes, clears, and creating offensive threats in the backcourt—indispensable components of competitive badminton performance. Future research should prioritize the development of RT protocols specifically designed to enhance upper-limb strength and power, including progressive resistance exercises targeting the shoulders, arms, and wrists. Such interventions would provide a more comprehensive understanding of RT’s role in advancing badminton-specific skills and improving overall performance.

Additionally, the variability in training designs and assessments highlights the need for standardized methodologies in future research. Establishing the optimal proportion of RT within an athlete’s total training volume, along with its frequency and cost-to-benefit ratio, is essential for maximizing training efficiency. Investigating the combined effects of various RT modalities, such as core and lower-limb explosive power training, may offer valuable insights for optimizing outcomes (Nirendan, 2023; Huang et al., 2023). Long-term interventions exceeding 12 weeks with structured progression are particularly recommended, as they are more likely to produce sustained improvements.

Furthermore, underexplored areas such as flexibility, reaction time, and coordination warrant greater attention to provide a comprehensive understanding of RT’s effects. These variables are critical for sustaining high-level performance during dynamic and unpredictable match scenarios. The integration of advanced monitoring tools, such as velocity-based resistance training (VBRT), can improve the precision of RT interventions, provide deeper insights into training outcomes, and support the development of individualized programming (Zhang et al., 2022).

Moreover, future research should aim to report both positive and null outcomes of RT interventions. Studies that observe no significant improvements in lower-limb strength or badminton-specific skills are equally valuable for identifying the boundaries of training effectiveness and understanding inter-individual variability. Transparent reporting of such findings can reduce publication bias and support the development of more refined, individualized training strategies. By addressing these gaps, future research can expand upon the foundational insights established in this review to develop badminton-specific RT strategies. Such strategies should aim to optimize physical performance, enhance skill development, and ultimately improve overall competitive success.

## 5 Limitations

While this review provides substantial evidence of the effects of RT on competitive badminton players, several limitations should be noted. Most included studies focused on male participants, with limited representation of female athletes. This restricts the generalizability of findings, as females may respond differently to RT due to physiological and hormonal differences. Additionally, the effects of RT on upper-limb strength, critical for badminton-specific skills such as smashes and clears, remain underexplored. Only one study indirectly assessed upper-limb strength, highlighting a key research gap.

Some studies lacked detailed descriptions of their RT protocols, including exercise progressions and compliance rates, which hinders reproducibility and evaluation of outcomes. Variability in RT modalities—such as training duration, frequency, and intensity—complicates the ability to generalize findings. Moreover, inconsistent reporting of performance assessments, particularly badminton-specific measures like smash velocity and shuttle speed, limits comprehensive analysis. The absence of control groups in certain studies further introduces bias and challenges the interpretation of RT’s true effects.

These limitations underscore the need for future research to employ standardized methodologies, include diverse participant groups, and provide detailed intervention protocols. Addressing gaps in upper-limb strength research and developing badminton-specific RT interventions will enhance the precision and applicability of findings.

## 6 Conclusion

This systematic review, encompassing 14 published studies, provides robust evidence that RT enhances physical fitness and skill-related performance in competitive badminton players. RT notably enhances explosive power, muscular strength, speed, and agility, which are critical for high-level badminton play. However, some important performance aspects, including upper-limb strength, flexibility, reaction time, and coordination, were underrepresented or rarely assessed, limiting the comprehensiveness of current evidence. Additionally, the optimal levels of maximal muscle strength necessary for badminton remain unclear, as excessive increases in muscle mass may negatively effect on-court efficiency. Future research should address these gaps by developing targeted RT programs tailored to badminton’s specific physical and technical demands, optimizing training effectiveness, and providing practical guidelines for elite badminton players and coaches.

## Data Availability

The datasets presented in this study can be found in online repositories. The names of the repository/repositories and accession number(s) can be found in the article/supplementary material.
